# Institutional Questions

**Published:** 1900-03-03

**Authors:** 


### INSTITUTIONAL QUESTIONS.

**The Mary Wardell Convalescent Home for Scarlet Fever.**

Note.—Under this heading a query was asked in this column a short time ago. We have received the following letter from Miss Mary Wardell, which will interest our correspondent to see. Miss Wardell writes:—

*** "In your issue of to-day (February 10th) you give a question respecting this home, and your reply. May I point out first that the term 'paying patients' is scarcely applicable to the women and children who are asked to contribute a fraction of their cost to the institution. Each patient costs the home from £2 10s. to £3 10s. a week. Secondly, male adult patients are admissible to the first-class department. I am only debarred from admitting male adults to the working-class department by not having received public support sufficient to enable me to build accommodation for them. Lastly, all inquiries should be addressed to myself, Miss Mary Wardell, Stanmore, and as being the hon. secretary, and not to the matron."

